# Winter-to-summer seasonal migration of microlithic human activities on the Qinghai-Tibet Plateau

**DOI:** 10.1038/s41598-020-68518-w

**Published:** 2020-07-15

**Authors:** Guangliang Hou, Jingyi Gao, Youcheng Chen, Changjun Xu, Zhuoma Lancuo, Yongming Xiao, Linhai Cai, Yuanhong He

**Affiliations:** 1 School of Geographic Science, Qinghai Normal University, Xining, 810008 China; 2 Academy of Plateau Science and Sustainability, Xining, 810008 China; 3 School of History, Capital Normal University, Beijing, 100089 China; 4 Key Laboratory of Geomantic Technology and Application of Qinghai Province, Xining, 810008 China; 5 Qinghai Provincial Institute of Cultural Relics and Archaeology, Xining, 810008 China; 6 Department of Archaeology, School of History & Culture, Sichuan University, Chengdu, 610000 China

**Keywords:** Climate-change impacts, Climate-change adaptation

## Abstract

The Qinghai-Tibet Plateau (QTP) has become a valuable site for investigation of adaptive regimes of prehistoric humans to extreme environments. At present most studies have focused solely on a single site. Using a more integrated approach that covers the complete scope of the plateau is needed to better understand the expansion logic of prehistoric humans moving towards the plateau. Here, we conducted accelerator mass spectrometry ^14^C dating of two microlithic sites. Canxiongashuo (CXGS) and Shalongka (SLK), which are located at the inner and marginal areas of the QTP, respectively. By using geographic information system, literature, and natural environmental factors, we constructed a model for the relationship between traveling distance and time, and we also used these factors to construct a plateau environmental index. The results indicated that the ages of the CXGS and SLK sites are 8.4–7.5 cal. ka BP and 8.4–6.2 cal. ka BP, respectively. Combining the archaeological evidence and literature, hunter-gatherers may have seasonal migration activities at low altitude in winter and high altitude in summer in order to make full use of natural resources. Our model of relationship between traveling distance and time shows that hunter-gatherers in CXGS site was active on the plateau all year-round at approximately 8.3 cal. ka BP. According to EI and archaeological remains, we propose that SLK site was a winter camp of prehistoric hunter-gatherers. Taken together, we determined 8.4–6.0 cal. ka BP as a transitional period from the Paleolithic to Neolithic Ages, and winter camps of hunter-gatherers evolved into settlements in the Neolithic Age.

## Introduction

There has been an increasing attention on the study of the activity patterns of hunter-gatherers during the transitional period between the Paleolithic and Neolithic Ages^1–3^, and it is generally considered that prehistoric human during that time are high in mobility. The Qinghai-Tibet Plateau (QTP) with its vulnerable environmental, is extremely sensitive to climate change^4^. Furthermore, scarce vegetation cover, low temperature, and dificient in oxygen atmosphere during the winter time, resulted in extremely harsh environment for prehistoric humans to settle^5,6^. Therefore, in academic community there is an increasing interest on the prehistoric huaman’s adaptation to the QTP and a striking progress has been made in the study of the prehistoric human–environment relationships on the QTP^7–10^. These studies provide insights to help us comprehensively understand the process of human-land relationship. Nevertheless, there are controversial opinions on the prehistoric humans’ occupation of the plateau due to the lack of surveys and excavations^11,12^.

Previous studies suggest that hunter-gatherers did not occupy the plateau during the winter until to the Neolithic age. It was not until 3.6 cal. ka BP with the support of animal domistication and barley cultivation that prehistoric humans could occupy the plateau all year round^13^. However, according to the studies of Chusang site there are some scholars believe that hunter-gatherers had occupied the plateau all through the year since the early Holocene^14^. Nevertheless, this conclusion has been questioned owing to the controversy over its travel cost modelling of hunter-gatherers^11^. At present, many studies showed that the life style of hunter-gatherers before the Neolithic Age was dominated by high mobility^15^. It is likely that they migrated to lower elevation regions in order to avoid extremely harsh winter environments, while they would have moved to higher elevation, regions to hunt in the summer^6,11^. Thus, to determine whether the prehistoric humans had occupied the QTP in winter was the resolution for this dispute. This is also a key step for prehistoric human to adapt the extreme environment condition of the plateau.

Over the past several decades, many microlithic sites of early-mid Holocene have been discovered on the QTP. Research shows that microlithic technologies were used by prehistoric humans^3^, who mainly hunted small- and medium-sized mammals, such as antelope, deer, and marmot, which had dominated the plateau during that period^16,17^. These hunter-gatherer activities had two characteristics: first, that their activities were relatively scattered, with long distances and frequent migrations; second, their camps were characterized by seasonality and randomness. There were additional sites with rich remains and multiple functions, such as those at the Heima River, Yantaidong, Jiangxigou 2, and Layihai, which may have been repeatedly and seasonally used for a long time as the sites had properties of central camps^18,19^. These findings suggest that large groups of hunter-gathers often came to Qinghai Lake Basin for long-term or seasonal living.

This seasonal migration pattern is also very common on the QTP even today^3^. In particular, this traditional nomadic seasonal migration between winter and summer camp is very popular today in the Qinghai Lake Basin (Supplementary Fig. 1). The vertical zonality of QTP leads to seasonal migration of wildlife between high and low elevations. Wild donkey is an excellent example. In winter, they tend to go from highland to low-elevation plain, while in summer, they go from low-elevation plain to highland. Thus, people in the Qinghai Lake Basin are also deeply influenced by the seasonal migration of wildlife and they perform regular seasonal migration pattern each year. More specifically, in winter, in the lowlands along the lake are the tribal settlements, because the highlands are extremely cold and the snow is deep, and it is difficult for livestock to get food, while during summer, people return to the river valley regions and move to the high elevation mountains. The low elevation pastures on the lakeside are reserved for their winter fodder. This seasonal migration has some typical characteristics: there are relatively fixed camps and routes across the four seasons and a one-way length is within 200 km. Notably, this migration has a seasonal property, and most of these routes are along rivers. The nomadic seasonal migration along the Haergai River can serve as an example. From November to April of the following year, people camped at Nalongwaerma, which is located at the ground level near the Qinghai Lake, was used as a winter camp, owing to its low elevation and rich hay. In the following April, people migrate to the spring camp, Qingdama, which has higher elevation. From June to July, they arrive at their summer camp, Mori in the river valley of the upper reaches of the Datong River. Finally, from September to October, they move to their autumn camp, Adasi. In November, they return back to their winter camp at Nalongwaerma. The entire one-way travel distance is approximately 110 km, which is repeated each year (Supplementary Fig. 1).

**Figure 1:**
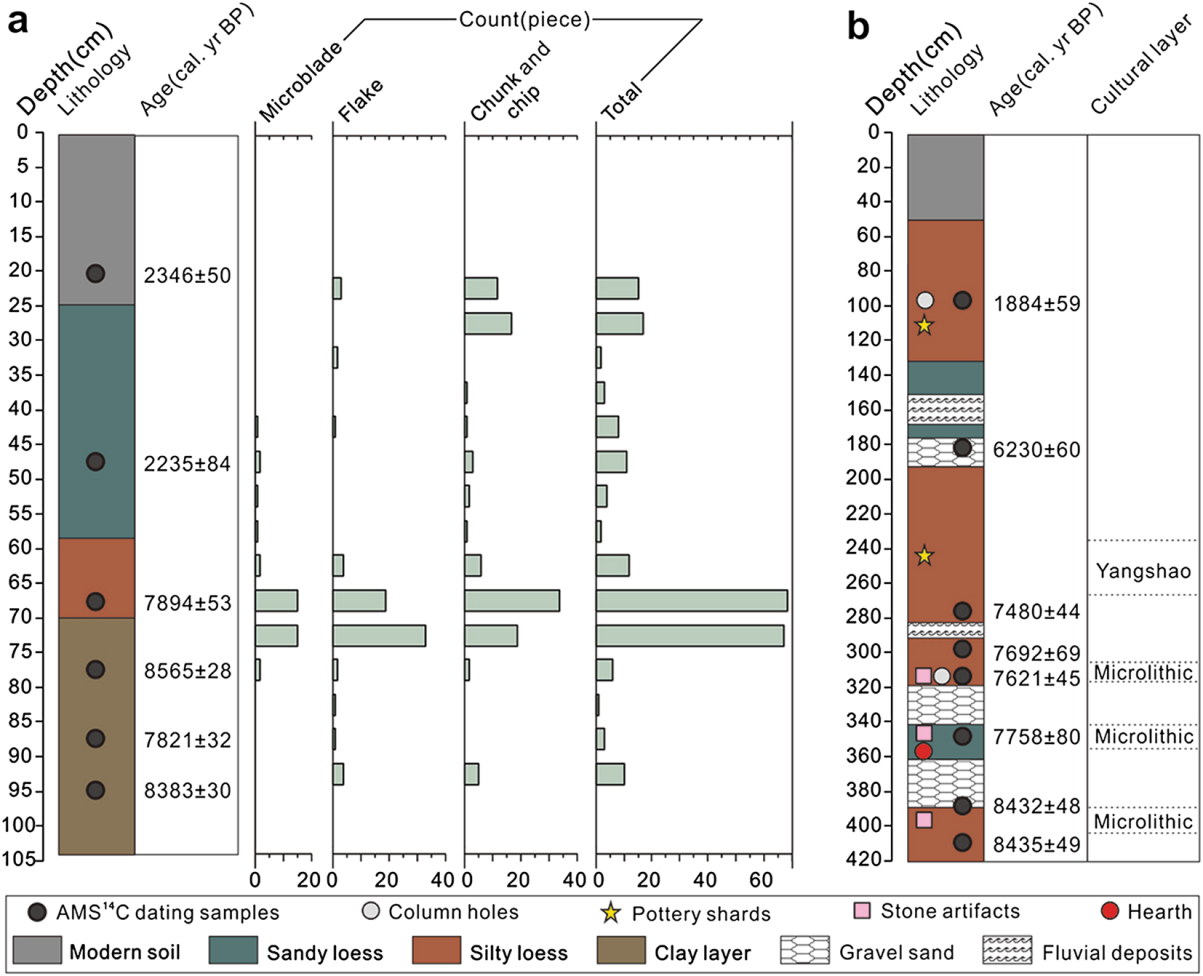
The stratigraphic lithology of sedimentary sequences and the positions of AMS ^14^C data and microliths in (**a**) CXGS and (**b**) SLK sites.

The above mentioned subsistence strategy of seasonal migration in the Qinghai Lake Basin is highly consistent with the ecological and phenological changes of the QTP^20^. Previous studies have suggested that the growth period of vegetation on the QTP starts from the end of April to the beginning of May, with the maturity period at the end of July to the end of August. At the beginning of September growth starts to decline, and ends at the middle-to-late October. Finally, the dormancy period starts in the middle of November and ends the following April. Based on the archaeological evidence and living data of modern residents^16–19,21^, this seasonal migration pattern seems to have obvious changes from the Last Deglacial to modern times, which is a relatively fixed life tradition. Given these findings, it can be used as a reference for understanding the patterns of prehistoric human activities.

In this paper, our research period is focused at 8.5–6.0 cal. ka BP of the early-mid Holocene. The accelerator mass spectrometry (AMS) ^14^C dating was employed in the Canxiongashuo (CXGS) (inner of the plateau) and Shalongka (SLK) (margin of the plateau) sites section. We used geographic information system (GIS) to establish a model for the relationship between traveling distance and time as well as the plateau environmental index (EI). The objective of the study was to provide chronological information and activity characteristics of CXGS and SLK sites, and determine whether the humans of the CXGS site occupied the plateau all year-round and the nature of the SLK site camp. On this base, this study further elucidated the distribution area of the winter camps on the QTP and the characteristics of the prehistoric human activities during the transitional period from Paleolithic to Neolithic Age.

## Results and discussion

### Age

CXGS section was approximately 105 cm thick and stone artifacts were found in a layer of 95–20 cm deep with most of them were found in layers of 70–65 and 75–70 cm deep. The AMS ^14^C dating results of CXGS section indicates stratigraphic deposition since the early-late Holocene, but the calibration ages of charcoal were notably inverted in 95–70 cm depth, which is common in the stratum affected by human activities. Basically, the calibration ages of the charcoal were mainly concentrated across two periods: (1) 8.4–7.8 cal. ka BP, the results of four charcoal ages occurred at a depth of 95–65 cm, and (2) 2.3–2.2 cal. ka BP, the results of two charcoal ages occurred in the stratum at a depth of 50–21 cm. In addition, the first occupancy of microlithic artifacts was at approximately 8.4 cal. ka BP and continued until 7.5 cal. ka BP. The number of microlith significantly intensified at around 7.8 cal. ka BP (Fig. 1a, Table 1).

**Table 1:** The AMS ^14^C dates from the CXGS and SLK sections.

Section	Lab number	Depth (cm)	Dating material	Radiocarbon age (year BP)	Calibrated age (2σ: cal. year BP)
CXGS	BA 151,596	21	Charcoal	2,330 ± 20	2,346 ± 50
	BA 151,597	45–50	Charcoal	2,210 ± 25	2,235 ± 84
	BA 151,598	65–70	Charcoal	7,055 ± 25	7,894 ± 53
	BA 151,599	75–80	Charcoal	7,765 ± 30	8,565 ± 28
	BA 151,600	85–90	Charcoal	6,985 ± 25	7,821 ± 32
	BA 151,601	95	Charcoal	7,570 ± 25	8,383 ± 30
SLK	BA 161,048	98	Charcoal	1940 ± 25	1884 ± 59
	BA 161,049	183	Charcoal	5,390 ± 45	6,230 ± 60
	BA 161,051	275	Charcoal	6,605 ± 30	7,480 ± 44
	BA 161,052	297	Charcoal	6,865 ± 30	7,692 ± 69
	BA 161,054	317	Charcoal	6,760 ± 30	7,621 ± 45
	BA 161,055	321	Charcoal	6,875 ± 30	7,723 ± 67
	BA 161,056	348	Charcoal	6,970 ± 30	7,758 ± 80
	BA 161,057	393	Charcoal	7,640 ± 30	8,432 ± 48
	BA 161,058	408	Charcoal	7,645 ± 30	8,435 ± 49

SLK section was approximately 415 cm thick. According to the unearthed cultural relics (pottery shards, stone artifact), the bottom-up cultural sequence was 413–304 cm as the microlithic culture layer, 280–250 cm as the Yangshao culture layer (6.0–5.0 cal. ka BP), 110–88 cm as the Qijia culture layer (4.3–3.6 cal. ka BP) and 88–78 cm as the Kayue culture layer (3.6–2.6 cal. ka BP). In addition, each cultural layer was mixed with different thicknesses of fluvial deposits. The dating results of the SLK section indicates stratigraphic deposition since the early-late Holocene, and had a deep section with several culture layers. These layers were also interactively deposited with fluvial deposit-silt layers (Fig. 1b, Table 1). The age-depth correlation was reasonably good and relatively continuous. In general, this stratigraphic deposition from 8.4 cal. ka BP. The period of microlithic hunter-gatherers was found at 8.4–6.2 cal. ka BP, and the peak of microlithic artifacts occurred in the stratum approximately 7.6 cal. ka BP. Furthermore, there were regular column holes in the stratum, which were speculated by the archaeological excavators to be closely related to architectural remains such as sheds.

By comparing the dating results and unearthed stone artifacts of the CXGS and SLK sites, it was determined that the prehistoric humans of the both sites all used microlithic technology. Also, these two sites were all dated from approximately 8.4 cal. ka BP and significantly increased at 7.8–7.6 cal. ka BP. Taken together, although the two sites were located in the inner and margin of the plateau, respectively, the nature and age of the microlithic hunter-gatherers at the two sites were basically similar.

### Traveling time in CXGS site

**Figure 2:**
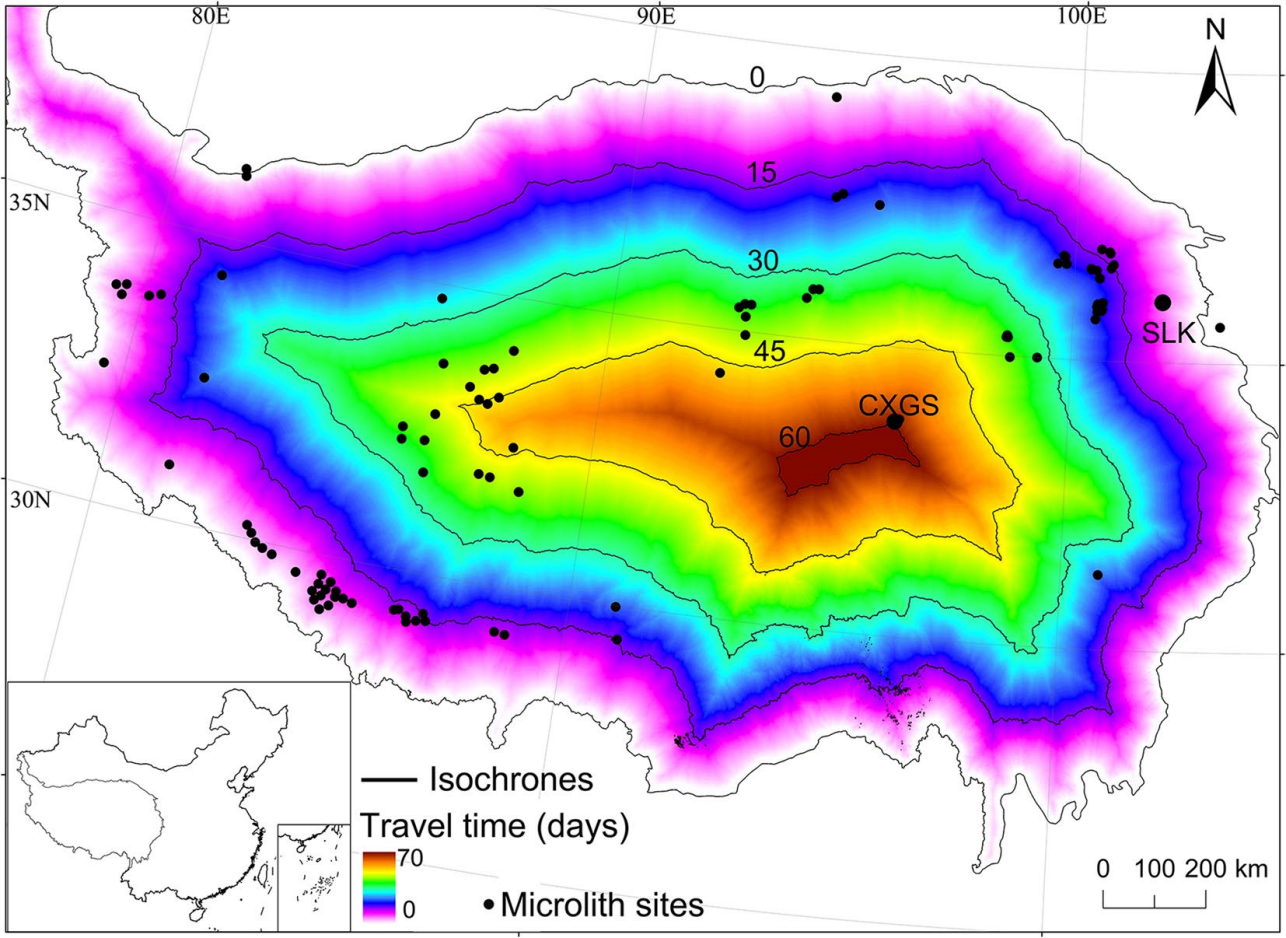
The map representing the relationship between distance and traveling time (days) from the margin to the inner of the plateau.

Based on the model of the relationship between traveling distance and time, we obtained analysis results of the relationship between traveling distance and time from the margin to the inner QTP (Fig. 2). These results showed that the time from the margin of the plateau to the other known microlithic sites on the plateau was less than 45 days^15–18^. However, it should be noted that the time from the margin of the plateau to the SLK site was less than 15 days but the time from the margin of the plateau to CXGS site was approximately 60 days (Fig. 2). As discussed above, the time for hunter-gatherers to arrive at the inner plateau during one year should not exceed 60 days. Thus, based on the behavioral patterns of the hunter-gatherers presented above, if any hunter-gatherers entered an area that took ≥ 60 days, they would be unable to leave the plateau. However, if hunter-gatherers from the margin of the plateau went to the CXGS site at the inner of the plateau, it would take approximately 60 days. The study suggests that due to the extreme environment (e.g., hypoxic conditions), travelling speed is affected not only by slope, but also by other factors such as elevation. Moreover, the decrease of maximal oxygen consumption (VO_2max_) and anaerobic threshold (AT) in the plateau environment results in decreased physical activity and labor^22^. In general, for every 1,000 m increase in elevation, labor endurance decreases by half. Therefore, the traveling speed in the plateau should be gradually lowered with an increase in elevation, resulting in increased travel time. In addition, hunter-gatherers in the plateau were mainly engaged in traveling, in which search of food and hunting activities played significant roles along the way. Additionally, during 8.5–6.0 cal. ka BP, there were no domesticated animals in the plateau to be used as transport tools, so hunter-gatherers in the plateau were slower than either those at lower elevations or those using animals for travel (e.g. documented sixteenth–twentieth century). Therefore, in the 60 days of traveling, the actual traveling distance was shorter than that calculated by the model. From the above analysis of simulation results, if the microlithic hunter-gatherers reached the CXGS site from the margin of the plateau in spring of a certain year during 8.3–7.5 cal. ka BP, it was difficult for them to get out of the plateau in autumn. In other words, both in summer and winter, the hunter-gatherers were active on the plateau and had not left the plateau that is, they occupied the plateau all year-round.

### Plateau environmental index and human activities

**Figure 3:**
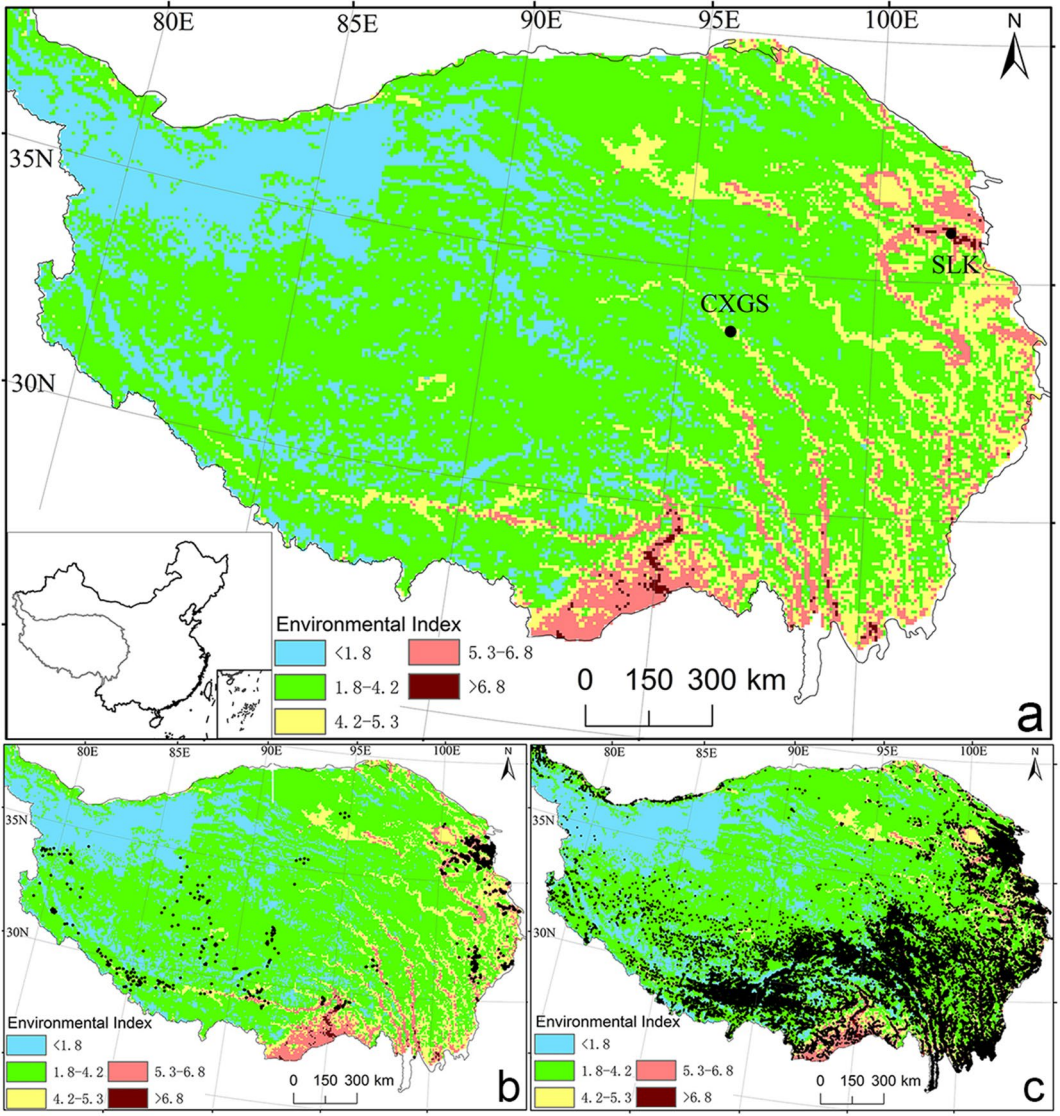
EI and settlements in the QTP (**a**) EI in QTP; (**b**) EI and Neolithic sites; (**c**) EI and modern settlements.

According to the above analysis, prehistoric hunter-gatherers in the inner of the plateau during early-late Holocene lived on the plateau year-round, specifically, they settled on the high elevation plateau in summer and low elevation plateau in winter. Here, the key question is which low elevation areas were suitable for the microlithic hunter-gatherer as winter camps? The research site of SLK in the study would be one of the alternative sites. There are two main reasons, first, this site had low elevation, high winter temperature, shallow snow, short snow-cover time, and as well as relatively superior environmental conditions. Furthermore, the deposition of the cultural layer in the microlith of the SLK section was not only thick, but continuous, in which many stone artifacts such as microliths were found. Especially, there was an emergence of several regular column holes in the culture layer in the SLK section’s microlith at a depth of 318 cm. Such column holes have been considered to be obvious signals of housing remains, indicating that prehistoric hunter-gatherers lived there for a long time, and there once was a relatively fixed seasonal camp with a long activity time and repeated use. Thus, we can infer that this site could be used as a winter camp for the microlithic hunter-gatherers. Also, it is implied that any site with environment conditions similar to the SLK site in prehistoric times may be used as a winter camp. In order to determine other natural environment areas similar to the SLK site, the plateau EI was constructed in this study (Fig. 3a). The results show that the EI of SLK site was 6.8. Thus, we defined areas with environmental indices of ≥ 6.8 as suitable areas for winter activity. It can be seen from Fig. 3 that the areas suitable for winter activity is mainly distributed in a few areas, such as Luoyu of the Yarlung Zangbo River, and the river valley areas of Guide and Minhe county at the upper reaches of the Yellow River.

On this basis, this study discusses the environmental index of Neolithic, Bronze Age and modern human settlements, as well as the distribution area of human activities related to it. During the middle Holocene (5.6–4.0 cal. ka BP), Neolithic Age cultures such as the Majiayao, Zongri, and Karuo were distributed within the plateau^23,24^ (Fig. 3b). Also, Fig. 3a or 3b show where these sites are located. Among these sites, houses have been commonly found, along with cultural relics such as ground stones and potteries, indicating that prehistoric hunter-gatherers had settled on the plateau. In particular, the Zongri site is mainly distributed at the upper reaches of the Yellow River, while the Karuo site is located at the upper reaches of the Lancang River on the inner plateau^24–26^. The EI of both two sites were approximately 5.3. Given this, areas with EI between 5.3 and 6.8 have been defined as areas for winter activity. Basically, such areas have been considered to be classic examples of settled areas on the plateau during the Neolithic Age, which mainly include the Shannan and river valley under the Qushui of Yarlung Zangbo River, the river valleys of three parallel rivers, Nujiang, Lancang and Jinsha Rivers, and the river valley area under Maqu of the upper reaches of the Yellow River. The Kayue culture (3.6–2.6 cal. ka BP) played an important role in the Bronze culture in the northeast of the QTP^13,24,27^. The introduction of a mixed economy of barley and animal husbandry finally enabled prehistoric human to permanently occupy on the inner part of the plateau^13^. This culture was mainly distributed in areas with an EI of ≥ 4.2; thus, we have defined areas with EI between 4.2 and 5.3 as transitional area of prehistoric hunter-gatherer activite area. Interestingly, the EI of modern settlements was found to be more than 1.8, while in the areas with EI of less than 1.8, there are less human activities, so they were defined as weakly active areas. According to the distribution pattern of Neolithic, Bronze Age and modern human, the areas with EI between 1.8 and 4.2 may be the areas where the prehistoric hunter gatherers active in summer. Therefore, it can be seen from Fig. 3 that the suitable areas for winter camps were mainly distributed in a few areas, such as the Luoyu region of the Yarlung Zangbo River, and the river valleys of the upper reaches of Yellow River in Guide County; And the prehistoric hunter gatherers were mainly active during the winter Shannan valley and the river valley under the Qushui of the Yarlung Zangbo River, the river valleys of three parallel rivers, Nujiang, Lancang, and Jinsha Rivers, and the river valley areas under the Maqu River of the upper reaches of the Yellow River. These analyses showed that suitable areas for winter camp and areas that were occupied by prehistoric hunter gatherers during the winter were basically river valley areas with relatively low elevation and good climate conditions.

**Figure 4:**
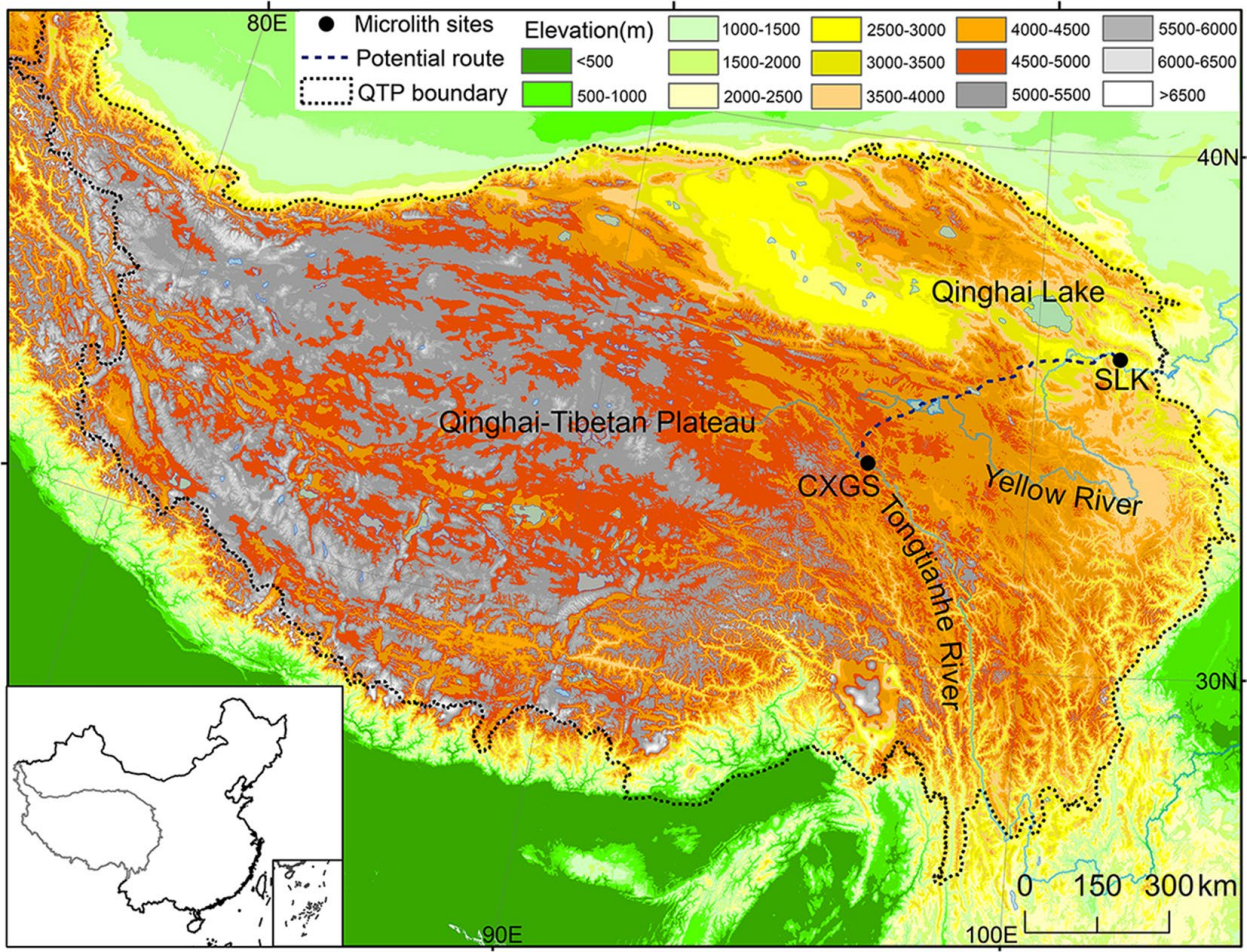
The topographic map of the QTP and the potential route between the SLK and CXGS sites. It is worth noting that the potential route is the shortest path based on slope simulation. Inset shows the location of the QTP in china.

It is worth noting that if only the environmental conditions are considered, the SLK site in this study may also be a winter camp for the prehistoric humans at the CXGS site (Fig. 4), however, if the long distance between the two sites, the large energy consumption, and the risk along the way are also considered, the SLK site is unlikely to be the winter camp for the prehistoric humans at the CXGS site. If the CXGS site is regarded as a summer camp, the corresponding winter camp is possibly distributed in the river valley of the lower reaches of the Tongtian River with warm and wet environment. More importantly, these two camps have a shorter distance, which needs approximately 15–35 days for prehistoric humans to arrive at one camp from another. In summary, prehistoric humans are likely to follow the “up and down the plateau” seasonal migration pattern along river valleys with better environmental conditions.

### Evolution and possible mechanisms from the microlith to Neolithic Ages

Some studies have revealed that the competitive pressure of the agricultural population in the Loess Plateau resulted in the appearance of specialized epi-Paleolithic blade and bladelet technologies on the high plateau after 8.2 cal. ka BP^28^. This may indicate more permanent occupation and new full-time residents on the plateau. Based on the above discussion, the CXGS site of the inner plateau and the SLK site of the marginal plateau are likely to be the classic examples of summer and winter camps related to the activities of microlithic hunter-gatherers on the plateau, which also support the view that after 8.2 cal. ka BP, there was emergence of hunter-gatherers active on the plateau all year-round. In addition, the history of human development indicates that in different periods, human activities developed characteristics of inheritance and continuity. The research period of this study was the transitional stage between the Paleolithic and Neolithic Ages on the plateau and the human behavior pattern that was the transitional stage from hunting and gathering to agriculture, from mobility to settling, and from seasonal camps to settled camps. These have clear signs of transition, inheritance, and continuity. Although we found a sedimentary layer mixed with proluvial and fluvial deposits in SLK section, the more obvious feature is that the Yangshao culture layer superimposed on the microlith culture layer, and the column holes representing human settlement behavior were found in the microlith culture layer (Fig. 1b), indicating that SLK site is probably evolved from the winter camp of microlithic hunter-gatherers to the settlement of Neolithic Age, and its potential distribution area is probably the suitable area for winter activities based on the plateau EI.

At 8.0 cal. ka BP, hunter-gatherers emerged were active on the plateau all year-round. This was likely owing due to the competition and population pressure of the agricultural people of lower elevations^29–31^, the applicability of microlithic technology and relatively superior natural environment conditions^32^. Specifically, the Dadiwan site of the Neolithic Age, which was dominated by agricultural cultivation, appeared in this period on the Loess Plateau (adjacent to the northeast of the QTP)^33^. Subsequently, hunter-gatherers were squeezed out of this area by the development of the Neolithic culture. In order to maintain the tradition of hunter-gatherers, prehistoric humans had to spread into the QTP all year-round on a large scale^6^. Due to the applicability of microlithic technology in the harsh environment, a large number of microlithic sites were found in the QTP during the middle Holocene, such as Jiangxigou 2^34^ and Yeniugou^35^. Additionally, quantitatively reconstructed precipitation based on pollen data from Lake Luanhaizi^36^, Lake Yidun^37^ and Lake Qinghai^38^ on the eastern QTP indicated that there was more precipitation in the early and middle Holocene^39^, less aeolian deposits weakly^40^, paleosols were formed mainly during 9.5–4.0 ka BP^41^. With the above background, QTP is likely to have seen the emergence of hunter-gatherers all year-round in the early and middle Holocene.

Surely, due to the complexity and particularity of human activities on the plateau, traveling speed and the time the hunter-gathers spent on the plateau depended not only on the slope, but also on related factors like elevation, topography, underlying surface, and weather. For example, hunter-gatherers were often confronted with glaciers, sand, swamps, and other underlying difficulties to pass. Large rivers and storms also became obstacles to traveling. Therefore, the actual traveling distance would be less than that calculated by our model regarding the relationship between distance and time. In other words, the hunter-gatherers’ traveling distance was less than the distance calculated by the model within the same time (60 days) spent on traveling.

## Conclusion

The CXGS site in the inner plateau and the SLK site on the margin of the plateau are all the remains of microlithic hunter-gatherers. The age of these microlithic hunter-gatherers was approximately 8.3–7.5 cal. ka BP and occurred in the early and middle Holocene. The age of the activities at the two sites is consistent with the relics excavated from the sites.

The microlithic sites on the plateau exhibited characteristics of dispersion, randomness, and seasonality. There should be seasonal migratory activities of microlithic hunter-gatherers between winter and summer camps. It means hunter-gatherers were active at low-elevation regions in the winter and at high-elevation regions in the summer. More specifically, hunter-gatherers moved to their high elevation summer camp from April to May, and returned to their low elevation winter camp from September to October. Furthermore, this seasonal migration between winter and summer needed to happen within no more than 60 days every year. According to our model regarding the relationship between distance and time at CXGS site, at 8.0 cal. ka BP, the microlithic hunter-gatherers did not leave the plateau in the winter. EI and archaeological evidence revealed that SLK site was a suitable winter camp for hunter-gatherers. Furthermore, the winter camp and distribution area of hunter-gatherers likely later evolved into a Neolithic settlement and distribution area of these sites.

## Materials and methods

### Study areas

The CXGS site (33°48′17″N, 96°02′36″E, 4,030 m a.s.l (above sea level)) is located on the first terrace of the Tongtian River in the center of the QTP (Supplementary Figs. 2a, 4). The accumulated temperature of ≥ 0 °C that is measured throughout the year (from the beginning of the farming period until to the start of grass growth) is 1,009 °C. This accumulated temperature can only develop independently to husbandry production (Table 2). There are only 20 frost-free days throughout the year when the daily minimum temperature is > 0 °C; these occur from July 24 to August 13. The vegetation is mainly alpine meadow, alpine grassland, and shrub. The CXGS site was first investigated and excavated in detail by the Institute of Cultural Relics and Archaeology of Qinghai Province. The CXGS site is located in the inner plateau and it is a large-scale site (1 km^2^) with the properties of a central camp. And a core area of approximately 15,000 m^2^. There are a large number of microblades, microblade cores, and other microlithic artifacts scattered on the surface of the site. Wedge-shaped microblade cores have long been considered a classic example of microlithic artifacts; notably, microblades and flakes also appeared at the site. Some studies have noticed that microblade technology in the CXGS site and "Layihai technology", which was found in the Yellow River Valley at the northeast margin of the plateau, exhibited a high degree of similarity in their microblade cores. This similarity is in terms of material selection, preform production, platform rejuvenation, and microblade detaching^42^, and indicates that central plateau had a close relationship with the plateau margin in their individual microlithic technologies. In the core area of the site, the Institute of Cultural Relics and Archaeology of Qinghai Province and other organizations established quadrats to excavate.

**Table 2:** Meteorological and industrial comparison between the CXGS and SLK sites. The CXGS and SLK sites data were from Zhiduo and Jianzha county meteorological stations whose distance were about 16 and 8 km during, respectively, 1960 and 1980.

Socioeconomic indicators	CXGS	SLK
Altitude (m a.s.l)	4,030	2076
Mean annual temperature (°C)	− 1.7	7.7
Mean January temperature (°C)	− 12.6	− 6.3
≥ 0 °C accumulated temperature (°C)	1,009	3,296
Mean annual precipitation (mm)	387	354
Mean January precipitation (mm)	1.8	2.1
Frost-free period of the year (day)	20	186
Mean annual snow depth (cm)	20	5
Mean annual snow days (day)	83	18
Industry	Planting industry	Animal husbandry

The SLK site (36° 02′ 52″ N, 101° 57′ 72″ E, 2076 m a.s.l.) is located on the second terrace of the Yellow River in the northeastern region of the QTP (Fig. 4, Supplementary Fig. 2c). The SLK site has been considered to be a suitable site for planting industry, owing to its accumulated temperature of ≥ 0 °C throughout the year. There are approximately 186 frost-free days throughout the year when the daily minimum temperature is > 0 °C; these occur from April 16 to October 19. In the past, the dominant vegetation was temperate forest and grassland; now, the vegetation is mainly cultivated plants, including spring wheat, broad bean and pea, and deciduous broadleaved trees such as poplar and willow (Table 2). The SLK site was first investigated and excavated in detail by the Institute of Cultural Relics and Archaeology of Qinghai Province. The Institute of Cultural Relics and Archaeology of Qinghai Province, which has carried out archaeological investigation, and found the area is approximately 24,000 m^2^, with complex cultural properties and continuous cultural sequences.

The CXGS site presents an extreme environment with low temperatures all year round. The winter features with deep and long term snow cover. In addition, severe disasters from snow and ice are prone to occur in the area. When compared with the CXGS site, the SLK site has much better conditions in the winter. There are no prominent meteorological disasters, making it a more suitable site to spend the winter time. However, there is a little difference in precipitation between the two sites.

### Sampling and dating

To obtain detailed ages of the prehistoric human activities in the CXGS and SLK sites, based on previous reports^43,44^, six pieces of charcoal were collected at depths of 21 cm, 45–50 cm, 65–70 cm, 75–80 cm, 85–90 cm, and 95 cm in the stratum of quadrats of the CXGS site, and nine pieces of charcoal were collected at depths of 98 cm, 183 cm, 250 cm, 275 cm, 297 cm, 310 cm, 317 cm, 321 cm, 348 cm, 393 cm, and 408 cm in the stratum of quadrats of the SLK site (Fig. 1b, Table 1). The charcoal samples collected from the above two sites were sent to the Quaternary Dating Laboratory of Peking University for AMS ^14^C dating. The AMS ^14^C dates were further converted to calendar year values by applying the IntCal 13 Calibration Curves using the Calib 6.1.0 program^45,46^.

### Data

The data used in this study were obtained mostly from the national geoscience data sharing platform (https://www.geodata.cn), including the scope and boundary of the QTP^47^, China Digital Elevation Model (DEM) of 1 km resolution (2000), China 1:250,000 data sets for hydrographic net classification of grades 1, 3, 4 and 5 (2002), China lake database of 1:100,000 (2000). Data regarding the accumulated temperature ≥ 0 °C used the database of the data level of temperature and humidity, which serves as the background level of Chinese ecological environment. This work was originally completed by the Institute of Agricultural Natural Resources and Agricultural Regionalization of Chinese Academy of Agricultural Sciences.

### Modeling the relationship between traveling distance and time

There were no domesticated animals during the period of time covered in this study, so there was no reliable animal power used during migration. As a result, travel relied solely on walking. Studies have shown that people will adjust their speed according to slope of the route in order to expend the lowest amount of energy on travel. Thus the size of a slope is a decisive factor for travel speed^48,49^. Traveling speed (v) and slope (p) have the following relationship^50^:

1 $$V = 5.1 \cdot \mathop {\text{e}}\nolimits^{{ - 2.25\left| {{\text{tg}}\left( {{\text{p}} \cdot \pi /180} \right) + 0.07} \right|}}$$

Then the time “t” required for traveling 1 m can be obtained from the following equation:

2 $${\text{t}} = 3600/\left( {1000 \cdot V} \right)$$

In Arc GIS 10.3, slope analysis was used to convert DEM of 1 km resolution of plateau into slope. In this study, raster calculator and formulae (1) and (2) were used to make cost raster data “t” for traveling distance in one second, taking CXGS site and “t” as the target layer and cost raster. The cost distance tool was then used to calculate the raster for the relationship between time and distance. Given this formula, the time spent going from the margin of the plateau to the inner of the plateau can be obtained. Similarly, the travel time from the margin of the plateau to the CXGS site can also be obtained, which was the cumulative time spent traveling from the nearest margin of the plateau to the CXGS site, in unit of “s”.

The key issue here is how much time did hunter-gatherers spend traveling each day? By referring to past historical records of traveling on the plateau (occurred between A.D. 1,620–1937, and each travel period was within one year)^51–53^, we attempted to answer this question. Specifically, the historical records counted the starting place, destination, route and time spent by past plateau travelers (Table 3). It is worth noting that, in comparison with the microlithic hunter-gatherers, past travelers using animal-powered travel had abundance of supplies for their journey and typically travelled with a clear direction. Contrastingly, constant search for food during the migration plays an important role in the travel of microlithic hunter-gatherers. As a result, unnecessary travel (in terms of their final destination) would be a large by-product of their final route. Previous traveler records indicate that a hunter's effective traveling time was approximately 4 h per day. In this paper, effective traveling time refers to the time from the starting place to the destination of traveling, without counting non-traveling time for other purposes. On this basis, the model of relationship between traveling distance and time was used to calculate the time from starting place to destination of the past travelers (Table 3). The comparison of historical records and simulation results showed that actual traveling time of past travelers was more than the simulated time, implying the average effective traveling time of past travelers on the plateau was no more than 4 h per day, with most traveling occurring in spring, summer, and autumn, while little traveling in winter. Thus, according to the above discussion, we assumed that there were seasonal camps for hunter-gatherers. They marched from winter camps to spring camps from April to May of each year, and were active at high elevation summer camps from June to August; and then they returned back to winter camps from September to October, and spend winter at low elevation winter camps from November to the next March. Overall, the time for departure and return was approximately 60 days, respectively each year, and the effective traveling time for daily was 4 h.

**Table 3:** The list of the traveling time spent by past travelers of the plateau.

Figure and event	Place of departure	Place of arrival	Traveling route	Travel time (days)	Simulated time (days)	References
Ma hetian investigates in Yushu	Xining	Yushu	Tang-Tibet ancient road	41	40.6	^43^
Missionary, Jean Grueber, into Tibet	Xining	Lhasa	Xining-Qaidam Basin-Lhasa	90	89.2	^44^
Missionary, Jean Grueber, out of Tibet	Lhasa	Nyalam	Not specified	30	23	^44^
Missionary, Francois Marley, into Tibet	Kathmandu	Lhasa	Passing through Nyalam -Dingri	60	34	^44^
Missionary, Dominic, into Tibet	Kathmandu	Lhasa	Passing through Nyalam	57	34	^44^
Missionary, Dominic, out of Tibet	Lhasa	Kathmandu	Passing through Nyalam	45	34	^44^
Missionary, Desideri, into Tibet	Leh	Lhasa	Passing through Qiangtang	223	96	^44^
Missionary, Desideri, into Tibet	Saga	Lhasa	Not specified	49	42	^44^
Missionary, Pedro Cabral, into Tibet	Bhutan	Shigatse	Not specified	33	12.5	^44^
The Northern route of Qing army's entering Tibet	Xining	Lhasa	Passing through the sources of the Yellow River and the Yangtze River	135	89.2	^45^
The northern route of Qing army's entering Tibet	Chengdu	Lhasa	Passing through Litang, Batang, Changdu	122	102	^45^

These events occurred in A.D. 1,620–1937, and each travel period was within 1 year.

### Constructed EI

To better elucidate the comprehensive natural environment conditions of the QTP, this study selected the vegetation type, elevation, river classification (river and lake), accumulated temperature of ≥ 0 °C, and longitude indicators as geographical and environmental factors based on the activity characteristics of the microlithic hunter-gatherers (Table 4). Using the reclassification and raster calculator tools in the spatial analysis of Arc GIS, we constructed an EI of the QTP to comprehensively characterize the natural environment of the QTP. Using this approach, a higher index would indicate better environmental conditions that are more suitable for human survival and living. The specific construction method is as follows:

**Table 4:** Classification and evaluation of geographical factors.

Typical types of vegetation	Preset value	Altitude (m)	Preset value	River	Preset value	≥ 0 °C accumulated temperature (°C)	Preset value	Longitude (°E)	Preset value
Temperate deciduous shruband, etc	9	< 1,600	9	Grade 1, 5 km	9	≥ 6,500	9	101.8–105.0	9
Temperate grass and forb meadow, etc	8	1,600–2000	8	Grade 1, 7.5 km	8	5,300–6,500	8	98.6–101.8	8
Temperate coniferous forests, subtropical and tropical mountain coniferous forests, temperate deciduous small-leaf forests, etc	7	2000–2,400	7	Grade 1, 10 km	7	4,200–5,300	7	95.4–98.6	7
Subtropical coniferous forests, broadleaf mixed forests, temperate deciduous broadleaf forests, etc	6	2,400–2,800	6	Grade 3, 5 km	6	3,500–4,200	6	92.2–95.4	6
Cold-temperate and temperate mountain coniferous forests, alpine meadow, etc	5	2,800–3,200	5	Grade 3, 10 km	5	2000–3,500	5	89.0–92.2	5
Subtropical evergreen broadleaf forests, etc	4	3,200–3,600	4	Grade 4, 5 km	4	1,500–2000	4	85.8–89.0	4
Subtropical evergreen broadleaf forests, etc	3	3,600–4,100	3	Grade 4, 10 km	3	1,000–1,500	3	82.6–85.8	3
Tropical rainforests, etc	2	4,100–4,600	2	Grade 5, 5 km	2	800–1,000	2	79.4–82.6	2
Dwarf trees desert, shrub desert, cushion subshrub Alpine desert, alpine dwarf semi-shrub alpine desert, alpine sparse vegetation, etc	1	4,600–5,500	1	Grade 5, 10 km	1	500–800	1	76.2–79.4	1
Alpine bog, desert, bare land, snow-capped land, saline soil, etc	0	> 5,500	0	> 10 km	0	< 500	0	73.0–76.2	0

Due to the diversity of vegetation types, only typical representatives were selected for “Typical types of vegetation” column heading. “River” Column heading represents grades 1, 3, 4, and 5 rivers, respectively; 5 km, 7.5 km, and 10 km represent buffer areas 0–5 km, 5–7.5 km, and 5–10 km from river, respectively.

Previous studies have shown that a mixture of forest-steppe was the dominated vegetation in which hunter-gatherers existed, followed by steppe^54^. Due to the vegetation density (forest) or relative lack of vegetation (desert), we concluded the sites that would not be conducive to human activities. The former would be difficult for prehistoric humans to conduct productive activities and transportation, while the latter would be more difficult to provide the necessary resources to support human societies owing to its inherent low biological productivity. Accordingly, the highest evaluation of temperature forests and steppe was 9, each type of temperature steppe was approximately 6–8, broad-leaved and coniferous forests were approximately 4–7, shrub was 6, meadow vegetation was 5, alpine vegetation and desert were approximately 1–2, and swamp and non-vegetation regions were 0.

In general, an elevation of 1,600–2,400 m a.s.l may result in a human response to the relatively low atmospheric oxygen. With an elevation above 3,000–3,600 m a.s.l, humans would have obvious signs of hypoxia. However, if humans are in an adaptive elevation ladder and have enough time, they will gradually adapt to these hypoxic plateau conditions. When the elevation is more than 4,500 m a.s.l and the atmospheric pressure is close to one-half of the sea level, humans will suffer from obvious hypoxemia and significant, negative physiological responses. When the elevation is higher than 5,500 m a.s.l, humans will suffer from a severe decline in function, with some damage being irreparable^55^. According to elevation, values from 0–9 were evaluated (Table 4), namely, elevation classification. With this ranking, lower evaluation numbers indicate elevations that are more difficult for humans to adapt to and survive in.

Rivers were divided into four grades 1, 3, 4 and 5 with each river used as a buffer area according to 5 km or 10 km distance from the main river. In general, the higher the grade is, the higher the evaluation of the region near the river.

The accumulated temperature of ≥ 0 °C plays an important role in characterizing the climate of the plateau. Specifically, plateau regions with accumulated temperatures ≥ 6,500 °C belong to tropical zones, < 500 °C belong to frigid zones, and approximately 1,500–4,200 °C represent temperate zones. At present, temperature crops are grown in areas of ≥ 2000 °C, while chimonophilous crops are exclusively grown in areas of < 2000°C^56^. Accordingly, the higher the accumulated temperature is, the higher the evaluation is.

Longitude is an important factor for prehistoric human to spread on the plateau. Since Last Glacial Maximum (LGM), the general direction of prehistoric human expansion to the QTP was a process going from east to west with the characteristics that the eastern sites have the early age results and the western sites have the late age results. The reasons for this expansion pattern are as follows. First, some scholars have proposed a “three-step jumping” model for human beings on their march into the plateau^57^. In this model, the first step was the LGM with an age range of 24.0–16.0 cal. ka BP. During this time, human activities occurred in the grassland at an elevation below 3,000 m. The second step was from 15.0–11.2 cal. ka BP during the Last Deglacial period, when hunter-gatherers expanded to regions with elevations of 3,000–4,000 m a.s.l. During this expansion, they left behind short-term camp sites, which were used to search for small- and medium-sized animals. The third and final step occurred during the early and middle Holocene when humans spread to high elevation regions above 4,000 m a.s.l. Second, genetic studies have revealed that approximately 98% of the maternal genetic component of modern Tibetans can be traced back to the northern Chinese who prehistorically moved into the QTP^6,58^. Tibetans have been recognized as Mongolians from East Asia, so there should be a view that people in northern China are expanded from east to west across the plateau^58^. However, the possibility of people originating from other directions and entering the plateau cannot be excluded, owing to the limitation of scale and differences in what time they may have entered the plateau^59^. Given this, these complexities have been omitted from the current discussion. Thus, we can conclude that the higher the longitude, the earlier the prehistoric age of human activity, and the higher the evaluation value.

Based on the above classification criteria and their application to the Analytic Hierarchy Process (AHP), each factor was evaluated, weighted, and the following relationship was constructed:

3 $${\text{I}} = 0.3 * {\text{H } + \text{ }}0.21 * {\text{R } + \text{ 0}}{.245} * {\text{P } + \text{ 0}}{.209} * {\text{T}}$$

In the formula (3), “I” represents the plateau EI, “H” is the elevation classification, “R” is the river classification, “P” is the vegetation classification, and “T” is classification of accumulated temperature of ≥ 0 °C. The higher the EI is, the more suitable it is for human survival and occupation. It should be pointed out that we used modern geographical factors to construct this EI, which are—to some extent—different from past environmental and geographical factors, but the changes of the natural environment are systematic and continuous, and the present natural environment is the inheritance of historical evolution, therefore the present natural environment has inseparable connections to its past form. Thus, the simulation used in this study plays an important role in such work.

## Supplementary information

Supplementary information

## Data Availability

All data needed for the evaluation of this paper are present in the paper or in supplementary information.
