# Author Correction: Winter-to-summer seasonal migration of microlithic human activities on the Qinghai-Tibet Plateau

**DOI:** 10.1038/s41598-020-75952-3

**Published:** 2020-10-29

**Authors:** Guangliang Hou, Jingyi Gao, Youcheng Chen, Changjun Xu, Zhuoma Lancuo, Yongming Xiao, Linhai Cai, Yuanhong He

**Affiliations:** 1 Academy of Plateau Science and Sustainability, Xining, 810008 China; 2 School of Geographic Science, Qinghai Normal University, Xining, 810008 China; 3 School of History, Capital Normal University, Beijing, 100089 China; 4 Key Laboratory of Geomantic Technology and Application of Qinghai Province, Xining, 810008 China; 5 Qinghai Provincial Institute of Cultural Relics and Archaeology, Xining, 810008 China; 6 Department of Archaeology, School of History and Culture, Sichuan University, Chengdu, 610000 China

Correction to: *Scientific Reports* https://doi.org/10.1038/s41598-020-68518-w, published online 15 July 2020

Yuanhong He was omitted from the author list in the original version of this Article. This has been corrected in the PDF and HTML versions of the Article, and in the accompanying Supplementary Information file.

The Author Contributions section now reads:

G.H. designed the study and supervised the field work. G.H., J.G., Y.X., L.C. and Y.H. conducted the archaeological investigations. Y.C. and Y.H. analyzed the stone artifacts. G.H., C.X. and Z.L. analyzed the data. G.H. drafted the initial manuscript, which was revised by G.H. and J.G. All authors approved the final version.

