# Supplementary material for: Winter-to-summer seasonal migration of microlithic human activities on the Qinghai-Tibet Plateau
Source: Sci Rep. 2020 Jul 15;10:11659. doi: 10.1038/s41598-020-68518-w (PMC7363859; doi:10.1038/s41598-020-68518-w)
Supplement: Supplementary file 1 — Supplementary information [file 41598_2020_68518_MOESM1_ESM.pdf]

Supplementary Information: Winter-to-summer seasonal migration of microlithic human activities on the Qinghai-Tibet Plateau

Guangliang Hou[1,2*], Jingyi Gao[1], Youcheng Chen[3], Changjun Xu[4], Zhuoma Lancuo[1], Yongming Xiao[5] & Linhai Cai[5], Yuanhong He[6]

[1] School of Geographic Science, Qinghai Normal University, Xining, 810008, China.

[2] Academy of Plateau Science and Sustainability, Xining, 810008, China.

[3] School of History, Capital Normal University, Beijing, 100089, China.

[4] Key Laboratory of Geomantic Technology and Application of Qinghai Province, Xining, 810008, China.

[5] Qinghai Provincial Institute of Cultural Relics and Archaeology, Xining, 810008, China.

[6] Department of Archaeology, School of History & Culture, Sichuan University, Chengdu 610000, China

*Corresponding author. E-mail: hgl20@163.com

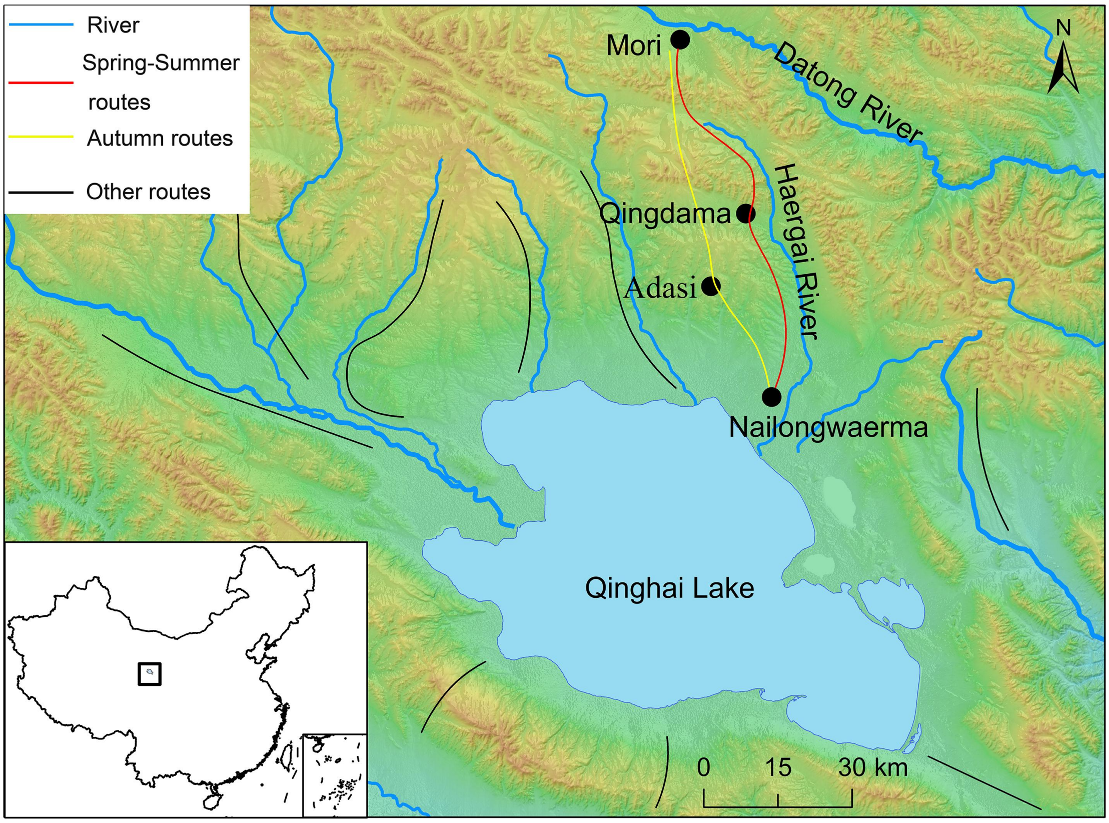

Supplementary Figure 1. Winter and summer migration routes in the Qinghai Lake Basin. This is an example of winter and summer seasonal migration on the QTP. Inset shows the location of the Qinghai Lake Basin in china.

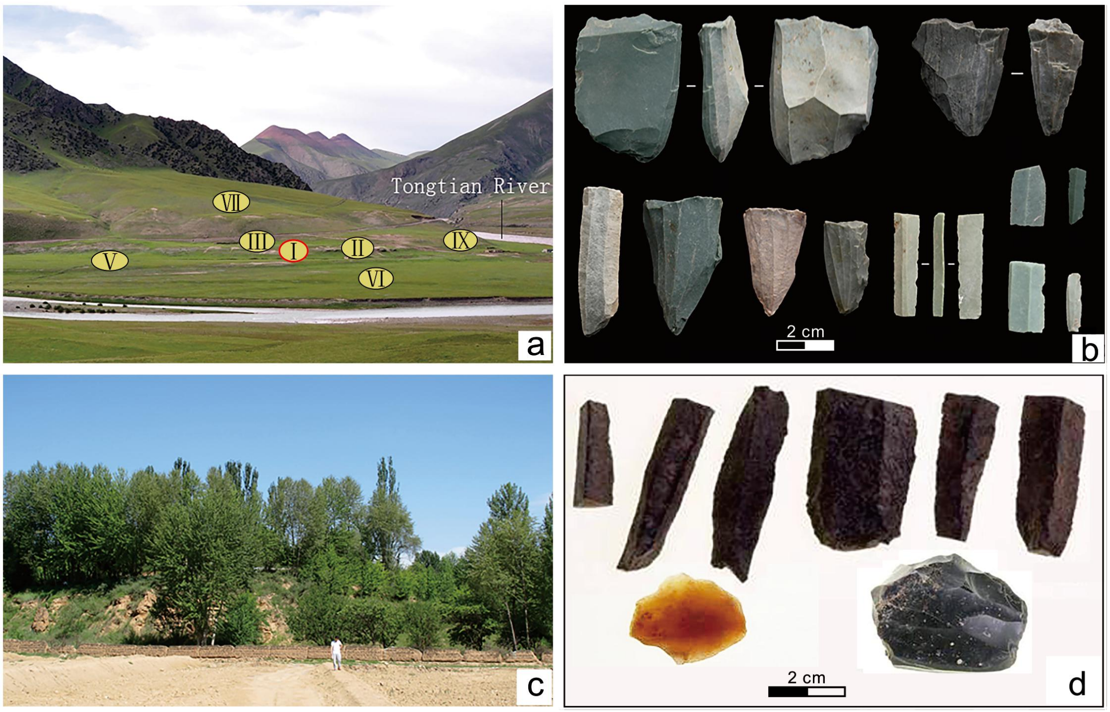

Supplementary Figure 2. Distant view and microliths of the CXGS and SLK sites (a) prospect in CXGS site; Ⅰ, Ⅱ, Ⅲ, Ⅳ, Ⅴ, Ⅵ and Ⅶ represent regional distribution of CXGS site by Qinghai Provincial Institute of Cultural Relics and Archaeology, and the CXGS section was selected from I area; (b) typical microliths were found in different depths of the CXGS site; (c) prospect in the SLK site; (d) microlith of the SLK site.
